# Perceptions and Interest in Lung Cancer Screening by Smoking Status: A Cross-Sectional Study of HINTS 6 (2022)

**DOI:** 10.3390/healthcare12191952

**Published:** 2024-09-30

**Authors:** Wenxue Lin, Ibrahim Alasqah, Saad A. Alotaibi, Nada Alqarawi, Sulaiman Sulmi Almutairi, Ariana Saraiva, António Raposo

**Affiliations:** 1 Department of Epidemiology and Biostatistics, College of Public Health, Temple University, Philadelphia, PA 19122, USA; 2 Department of Psychiatric and Mental Health, and Community Health, College of Nursing, Qassim University, Buraydah 51452, Saudi Arabia; i.alasqah@qu.edu.sa (I.A.); n.alqarawi@qu.edu.sa (N.A.); 3 Department of Public Health, College of Applied Medical Sciences, Qassim University, Buraydah 51452, Saudi Arabia; s.alrowes@qu.edu.sa; 4 Department of Health Informatics, College of Applied Medical Sciences, Qassim University, Buraydah 51452, Saudi Arabia; ssmtiery@qu.edu.sa; 5 Department of Animal Pathology and Production, Bromatology and Food Technology, Faculty of Veterinary, Universidad de Las Palmas de Gran Canaria, Trasmontaña s/n, 35413 Arucas, Spain; ariana_23@outlook.pt; 6 CBIOS (Research Center for Biosciences and Health Technologies), Universidade Lusófona de Humanidades e Tecnologias, Campo Grande 376, 1749-024 Lisboa, Portugal

**Keywords:** HINTS, LDCT, lung cancer, telehealth care, smokers, tobacco

## Abstract

Background: Lung cancer screening guidelines prioritize individuals with a history of smoking due to their higher risk of the disease. Methods: Our study examines the awareness and interest in low-dose computed tomography (LDCT) lung cancer screening among different smoking statuses using data from the National Cancer Institute’s Health Information National Trends Survey (HINTS) 6 (2022). We analyzed data from HINTS 6, including 3915 participants on smoking status, LDCT screening, and telehealth use. Participants were categorized as current smokers, former smokers, and non-smokers. Results: Current smokers had the highest likelihood of being recommended for LDCT screening (OR: 7.1, aOR: 10.4) compared with non-smokers. Former smokers also had increased odds of screening recommendations (OR: 3.1, aOR: 3.4) than non-smokers. Despite higher screening recommendations, current smokers exhibited significantly lower interest in cancer screening (interest rating score: 2.1) compared with non-smokers (interest rating score: 2.4) and former smokers (interest rating score: 2.5). Current smokers rated their telehealth care experiences more positively in terms of care quality compared with non-smokers. Conclusions: Our findings underscore a gap in cancer screening interest among current smokers despite their higher likelihood of being recommended for LDCT screening. The favorable perception of telehealth among current smokers provides an opportunity to enhance engagement and promote LDCT scan through telehealth care.

## 1. Introduction

Low-dose computed tomography (LDCT) is recommended by the CDC and the US Preventive Services Task Force for adults who are current smokers, former smokers, or senior adults older than 50 [1]. LDCT uses a small amount of radiation to obtain detailed images of the lungs without invasion or pain, which can be performed quickly [1]. The National Lung Screening Trial (NLST) demonstrated significant reductions in lung cancer mortality among current and former smokers screened by LDCT compared with those screened with chest radiography [2]. Further, a meta-analysis conducted by Hoffman et al. concluded that LDCT benefits in reducing lung cancer mortality while maintaining a low risk of false positives, screening complications, and overdiagnosis [3].

In 2021, the prevalence of cigarette smoking decreased to 11.5% from 20.9% in 2005 [4,5,6], but it continues to be the primary contributor of preventable diseases and deaths in the United States, causing more than 480,000 deaths each year [5]. Cigarette smoking is the primary risk factor for lung cancer, with over 80% of lung cancer deaths attributed to smoking [5,7]. Cigarette smoke contains at least 7000 toxic chemical compounds, over 70 of which are known carcinogens [5]. More than 28.3 million smokers face an elevated risk of smoking-related diseases, such as lung cancer [5,8]. There is a dose–response relationship between smoking behavior and lung cancer mortality; the longer a person smokes and the more cigarettes smoked per day, the higher the cancer mortality risk [7]. Smoking cessation is the best way to reduce lung cancer risk, and quitting at any age benefits smokers’ health by lowering cancer risk [7]. The menthol ban and Reduced Nicotine Content (RNC) cigarettes are two strategies under consideration by the United States Food and Drug Administration (FDA) to address the serious situation posed by cigarette smoking [9]. Studies have shown that RNC plays an important role in reducing blood cotinine levels, daily cigarette consumption, and tobacco toxicant exposure [9,10,11,12,13,14,15,16,17,18,19]. Meanwhile, a study has revealed that smokers with a desire to cease smoking were more inclined to believe in tobacco cessation medications [20].

Telehealth, initially designed to deliver basic care to individuals in rural or underserved areas [21,22], has experienced rapid expansion due to the COVID-19 pandemic [23,24,25]. Compared with traditional care, telehealth saves patients’ cost and time on transportation, decreases medication misuse and unnecessary visits, makes accessing care easier with smartphones or laptops with cameras, addresses provider shortages, and results in high patient satisfaction [21,25,26]. Past research has suggested increased telehealth usage rates across US populations even in post-pandemic care [27,28]. However, Everson et al. indicated that elderly individuals with limited internet access and lack of a college-level education were less likely to be offered telehealth care, although there was no difference in telehealth care usage once they had the opportunity [27]. Potential biases from healthcare providers’ expectations of the target population on telehealth might explain this phenomenon [27].

Cigarette smokers experiencing poverty encounter significant health disparities due to their exposure to nicotine and toxic chemicals [29], increased lung cancer risk, limited access to healthcare facilities, and targeting by tobacco industries [30]. The coronavirus disease 2019 (COVID-19) has unequally impacted socioeconomically underprivileged populations, including tobacco users with chronic diseases and those with low incomes [31,32]. Several studies have indicated disparities in telemedicine access by age and socioeconomic status (SES) [33], particularly showing that populations aged 65 years or older have a reduced use of telemedicine [34]. Most telemedicine was implemented by younger individuals with higher SES [35,36,37,38]. As a result, vulnerable populations, such as smokers with low SES or patients with chronic diseases from low SES backgrounds, may not use telemedicine frequently. Most of the research conducted thus far has focused on the health impact of smoking behavior, addiction, and smoke toxicants on tobacco users. While LDCT scans can significantly reduce lung cancer mortality, few studies have assessed smokers’ awareness and interest in LDCT scans, knowledge, preferences, and concerns about using telehealth care. Therefore, our study aims to investigate and compare the awareness and interest in LDCT scans by smoking status (former, current, and non-smokers). Additionally, we identify and compare the potential barriers to using telehealth between cigarette smokers and non-smokers.

## 2. Materials and Methods

### 2.1. The HINTS 6 (2022)

We used data from the National Cancer Institute’s Health Information National Trends Survey (HINTS) 6, a nationally representative survey conducted by the National Cancer Institute (NCI) since 2003 [39]. HINTS regularly collects data from all adults (aged 18 years or older) residing in non-institutionalized civilian households in the United States [39]. The survey aims to assess health-related information, behaviors, perceptions, and knowledge among US adults. HINTS 6, conducted from 7 March 2022 to 8 November 2022, used a multi-mode approach (paper and web modes) [39]. Participants were randomized into either Concurrent (paper and web modes) or Sequential (first web, then paper mode) groups for a mixed-mode experiment [39]. Detailed methodology and survey experiments are available on the NCI website (https://hints.cancer.gov/ (accessed on 8 July 2024)) and published elsewhere [39].

From the final sample of 6252 participants in HINTS 6, we excluded those with incomplete information on telehealth use (n = 206), discussion on LDCT scans (n = 628), smoking status (n = 378), and other variables (n = 1125). A total of 3915 participants were eligible for our analysis, comparing awareness and interest in LDCT scans for lung cancer among current smokers, former smokers, and non-smokers. Among these eligible participants, 11.3% (weighted proportion; unweighted n = 431) were current smokers, 22.2% (weighted proportion; unweighted n = 968) were former smokers, and 66.5% (weighted proportion; unweighted n = 2516) were non-smokers.

### 2.2. Measures

Participants were categorized based on their smoking status: current smokers, former smokers, and non-smokers. Non-smokers had smoked fewer than 100 cigarettes in their lifetime, former smokers had smoked more than 100 cigarettes but did not currently smoke, and current smokers had smoked at least 100 cigarettes and currently smoked (either every day or some days) [40,41,42].

We collected demographic information including age (continuous), race/ethnicity (Hispanic, Non-Hispanic White, Non-Hispanic Black, Non-Hispanic Asian, and Others), educational attainment (less than high school vs. post high school training/college), gender (male vs. female), employment status (employed vs. unemployed), and annual household income (≤$49,999 vs. ≥$50,000).

We examined LDCT lung cancer screening, interest in cancer screening, and telehealth care as outcomes. LDCT screening information was obtained from the question: “At any time in the past year, did a doctor or other health professional talk with you about having a low-dose CT (LDCT) scan to check for lung cancer?”. Responses were dichotomized as Yes vs. No, excluding “Don’t know” responses. Interest in cancer screening was measured by asking, “How interested are you in having a cancer screening test in the next year?” with responses ranging from 1 (not at all) to 4 (very). Responses of “Not applicable” or “I am up-to-date with screening tests” were excluded from our analysis. Telehealth care usage was assessed with the question: “In the past 12 months, did you receive care from a doctor or health professional using telehealth?”. Responses were dichotomized as Yes (via video or phone call) vs. No.

For those who received telehealth care, we collected responses to three follow-up questions regarding technical problems, quality of care, and privacy concerns. Responses were initially rated on a scale from 1 (strongly agree) to 4 (strongly disagree) but then reversed for simplicity of interpretation (1—strongly disagree, 2—somewhat disagree, 3—somewhat agree, and 4—strongly agree).

### 2.3. Statistical Analysis

We calculated descriptive statistics including unweighted counts, weighted means (for continuous variables), standard errors, and proportions (for categorical variables). We used the Rao–Scott chi-square test and *t*-test as appropriate [27,40,43]. Weighted logistic regression analysis estimated the odds of being recommended for LDCT screening based on smoking status. Both adjusted and unadjusted models were reported, with crude and adjusted odds ratios (aORs), 95% confidence intervals (CIs), and *p*-values. Weighted linear regression was used to estimate interest in cancer screening and assess responses to follow-up questions about telehealth care. Demographic information was included in adjusted models to control for potential confounders.

Statistical analyses were performed using R statistical software (version 4.3.3) with relevant packages (haven, dplyr, survey) to accommodate Taylor Series linearization sample weights as recommended by HINTS [27,44]. All tests were two-sided, with a significance level set at 0.05.

## 3. Results

Table 1 presents the characteristics of the study participants by smoking status. Current smokers were older (49.4 vs. 46.6 years), were predominantly Non-Hispanic White (72.8% vs. 67.5%), had lower educational attainment (less than high school: 38.4% vs. 26.3%), and a higher proportion had an annual household income between $0 and $49,999 (54.2% vs. 35.2%) than non-smokers (*p* < 0.05). They were also more likely to be unemployed (27.8% vs. 14.7%) and to be recommended for LDCT screening (10.8% vs. 1.7%) compared with non-smokers (*p* < 0.05). There were no significant differences in gender and the likelihood of receiving telehealth care among current, former, and non-smokers (*p* > 0.05).

Table 2 shows the weighted logistic regression analysis for the odds of being recommended for an LDCT scan by smoking status. Current smokers had higher odds (OR: 7.1, 95% CI: 4.3, 11.6; aOR: 10.4, 95% CI: 5.2, 20.8; *p* < 0.001) and former smokers also had higher odds (OR: 3.1, 95% CI: 1.8, 5.4; aOR: 3.4, 95% CI: 1.9, 6.1; *p* < 0.001) than non-smokers of being recommended to have an LDCT scan for lung cancer in both crude and adjusted models.

Table 3 presents interest in cancer screening by smoking status. Former smokers and non-smokers expressed some interest (interest rating: 2.5 and 2.4, respectively) while current smokers had significantly lower interest (interest rating score: 2.1, estimate: −0.2, *p* = 0.05) compared with non-smokers.

Table 4 details responses from telehealth users. Participants reported mostly no technical problems with telehealth visits, with no significant differences in smoking status (*p* > 0.1). Current smokers rated telehealth as good as in-person visits (rating score: 3.4) more compared with non-smokers (rating score: 3.0) in both crude (estimate: 0.4, *p* = 0.002) and adjusted (estimate: 0.3, *p* = 0.04) models. Privacy concerns were generally low, with no significant differences by smoking status (*p* > 0.05).

## 4. Discussion

Our findings indicate significant differences in LDCT lung cancer screening awareness and interest based on smoking status. Specifically, current and former smokers showed a higher likelihood of being recommended for LDCT screening compared with non-smokers. This is consistent with clinical guidelines that prioritize high-risk populations, such as current and former smokers, for lung cancer screening due to their elevated risk of developing lung cancer. Current smokers had the highest odds of being recommended for LDCT screening (OR: 7.1, aOR: 10.4), followed by former smokers (OR: 3.1, aOR: 3.4), underscoring the targeted approach in clinical practice to encourage screening among those at greatest risk. However, in terms of interest in cancer screening, former smokers and non-smokers expressed moderate interest, whereas current smokers showed significantly lower interest despite their higher risk of developing lung cancer. The lower interest among current smokers (interest rating score: 2.1) highlights a critical gap in public health outreach and education efforts aimed at encouraging this high-risk group to participate in screening.

Lung cancer screening is critical for early diagnosis and treatment, which contributes to a higher survival rate [45]. However, in 2021, only 5.8% of eligible Americans were screened for lung cancer, with the highest rate in Massachusetts at 16.3%, and the lowest rates in California and Nevada, at 1.0% and 1.3%, respectively [46]. More than 40% of lung cancer cases are diagnosed at a late stage, where the survival rate is less than 9% [1]. High-risk populations, such as smokers aged 50–80 years with a 20-pack-year history, current smokers, or former smokers, could reduce lung cancer mortality by up to 20% with annual low-dose CT (LDCT) scans [1,45,47]. In terms of age comparison, older smokers (≥55 years) might be more interested in cancer screening, as a prior study indicated that they are concerned about the risks of lung cancer, with nearly 80% of the study sample favoring LDCT [48]. Although the cancer mortality rates have decreased sharply by up to 31% due to reduced smoking prevalence, advancements in treatment, and early screening, lung cancer continues to pose significant challenges [49]. Smoking is responsible for over 90% of all lung cancer deaths, and more women die from lung cancer than from breast cancer [5,50,51]. The prevalence of current cigarette smoking among women is 10.1%, which is lower than the 13.1% prevalence among men [52]. Despite improvements in the five-year survival rate for lung cancer due to advancements in treatment, research, and clinical trials, the extremely low lung cancer screening rates remain a significant public health challenge. Over 14 million Americans are eligible for cancer screening [45]. The FDA’s strategies, such as reducing nicotine content (RNC) and implementing a menthol ban, are vital for tobacco control [43]. However, increasing awareness, interest, and perception of the importance of LDCT scan screening for lung cancer also plays a critical role in reducing mortality and improving health outcomes for smokers. Insufficient knowledge or perceptions of LDCT scan screening for lung cancer might further exacerbate health disparities, especially among smokers from low socioeconomic backgrounds, or people of color [45]. These populations often face limited access to healthcare, lower survival rates, delayed diagnosis, and reduced likelihood of receiving surgical treatment, ultimately leading to higher mortality of untreated lung cancer [45].

Approximately 37% of US adults used telehealth in 2021, according to the National Health Interview Survey (NHIS) [28]. In our study, we found that around 40% of participants received telehealth care regardless of their smoking status, which is consistent with the prior NHIS study and another study using HINTS 6 (2022) [27,28]. Several factors contributed to the popularity of telehealth, including consultation with healthcare providers, recommendations for telehealth care, flexibility in telehealth delivery, minimal risk of infection from in-person visits, and the hesitance to use paid leave [27]. Further, Everson et al. indicated that participants who chose telehealth care considered it as good as in-person care, with few perceiving it as a barrier to the care they received [27]. Our findings were similar, as most participants were in favor of the quality of telehealth care. Interestingly, there were no significant differences in the likelihood of receiving telehealth care among current, former, and non-smokers, suggesting that telehealth usage is relatively consistent across smoking statuses. However, current smokers rated their telehealth experiences more favorably compared with non-smokers, particularly regarding the perceived quality of care being as good as in-person visits. This positive perception among current smokers could be used to increase engagement with healthcare providers and promote discussions about lung cancer screening during telehealth visits.

Therefore, the integration of telehealth into routine healthcare services offers a significant opportunity to address the low screening rates for lung cancer, particularly among cigarette smokers who are at higher risk. Our study indicates that telehealth services are favorably received by current smokers, with many rating their telehealth experiences as good as in-person visits. Further, given the minimal risk of infection and the flexibility offered by telehealth, it is an appealing option for smokers who might otherwise avoid in-person visits. This is particularly important for those who are less interested in lung cancer screening or are reluctant to use paid leave for medical appointments. By emphasizing the convenience and quality of telehealth care, doctors, clinicians, or public health professionals can better reach this high-risk group and encourage the use of LDCT scans for the early detection of lung cancer.

Our study has several limitations. First, we focused on combustible cigarette users; however, electronic cigarette users, or e-cigarette users, represent another emerging public health issue. The health impact of e-cigarettes is not clearly known, as they have only been on the US market since 2006, and there has not been enough time for long-term health assessments for vapers [53]. Second, HINTS targets US adults, which means the associations we observed may have limited external validity (generalizability) to smokers residing in countries other than the United States. Third, those who have smoked fewer than 100 cigarettes in their lifetime may not necessarily be true non-smokers, as our definition could include individuals who are currently smoking or occasionally smoke cigarettes [54]. Further, smoking status could be determined more precisely if we had information on the number of cigarette packs smoked. Forth, our study focused only on US adults and did not include adolescent smokers. These youth smokers may also need lung cancer screening tests in the future as they may consume more cigarettes as they grow older. Another future study could involve investigating the presence of systemic diseases and their relationship to visits to a doctor, who in turn recommends LDCT screening. Further, it is important to note that a high perception of telemedicine use does not necessarily mean participants will actually undergo a CT scan, and it does not mean that patients who initially showed no interest in LDCT will not eventually get one. Given the nature of the cross-sectional study design, it is unlikely that our study could capture such information. Therefore, we need to interpret the association observed in our study with caution, given the potential limitations in establishing a connection between telemedicine use and actual CT scan visits. More importantly, the HINTS 6 survey was not designed specifically for drawing inferences on lung cancer screening based on participants’ responses. This may introduce several biases, including incomplete responses, low response rates, and potential measurement errors or misclassification. Another important variable, such as comorbidities, was not included in our study. Patients with chronic conditions may be more likely to seek or receive regular medical attention, which could contribute to lung cancer screening and prophylaxis. Our study did not account for these variables. Potential reasons for low interest in lung cancer screening could include low socioeconomic status, educational attainment, lack of insurance, income issues, or simply fear of negative scan results. Our future study will explore these factors and aim to find potential solutions to increase interest in lung cancer screening among smokers. It is crucial to understand why some participants have limited interest in or access to telehealth. Factors such as age, socioeconomic status, educational attainment, income, smoking status, or other potential factors could contribute to reduced use of telehealth. Understanding these reasons could be very helpful for clinicians in the ongoing development and improvement of telehealth services.

## 5. Conclusions

In conclusion, our study highlights significant disparities in lung cancer screening awareness and interest among different smoking statuses. While current and former smokers are more likely to be recommended for LDCT screening, current smokers show lower interest in participating in such screenings. The favorable perception of telehealth among current smokers provides an opportunity to enhance engagement and promote secondary prevention through telehealth platforms. Incorporating telehealth into public health strategies may help to increase awareness, interest, accessibility, and the use of lung cancer screening, improving early detection rates and reducing lung cancer mortality among smokers.

## Figures and Tables

**Table 1:** Characteristics of participants by smoking status, HINTS 6 (2022).

	Current Smokers n = 431 (11.3%)	Former Smokers n = 968 (22.2%)	Non-Smokers n = 2516 (66.5%)	*p*-Value
Gender				0.235
Male	179 (53.5)	451 (51.2)	1001 (48.1)	
Female	252 (46.5)	517 (48.8)	1515 (51.9)	
Race/ethnicity				0.011
Hispanic	12 (1.1)	31 (2.4)	170 (5.5)	
NH *-White	287 (72.8)	748 (79.4)	1619 (67.5)	
NH-Black	91 (13.7)	116 (9.4)	419 (12.4)	
NH-Asian	14 (6.7)	27 (2.2)	164 (7.6)	
Others	27 (5.7)	46 (6.6)	144 (7.0)	
Education				0.004
<High school	149 (38.4)	229 (28.7)	455 (26.3)	
≥High School	282 (61.6)	739 (71.3)	2061 (73.7)	
Annual household income				<0.001
$0 to $49,999	265 (54.2)	396 (34.5)	919 (35.2)	
≥$50,000	166 (45.8)	572 (65.5)	1597 (64.8)	
Employment status				<0.001
Employed	212 (72.2)	436 (81.1)	1545 (85.3)	
Unemployed	219 (27.8)	532 (18.9)	971 (14.7)	
Received telehealth care				0.5
Yes	164 (39.8)	426 (41.7)	1027 (38.2)	
No	267 (60.2)	542 (58.3)	1489 (61.8)	
Discussed low-dose CT for lung cancer				<0.001
Yes	54 (10.8)	57 (5.1)	39 (1.7)	
No	377 (89.2)	911 (94.9)	2477 (98.3)	
Age, year	49.4 (14.7)	56.9 (16.2)	46.6 (18.2)	<0.001

Data source: HINTS 6 (2022). Categorical variables: unweighted N (weighted %); Continuous variables: Mean (SE). *p* value was calculated by the Rao–Scott x2 test and *t*-test for categorical variables and continuous variables, respectively. * NH: Non-Hispanic.

**Table 2:** Crude and adjusted logistic regression models estimating association between recommended for LDCT scan for lung cancer and smoking status.

	Non-Smokers	Former Smokers OR (95% CI)	*p*-Value	Current Smokers OR (95% CI)	*p*-Value
LDCT scan to check for lung cancer					
Crude	Ref	3.1 (1.8, 5.4)	<0.001	7.1 (4.3, 11.6)	<0.001
Adjusted *	Ref	3.4 (1.9, 6.1)	<0.001	10.4 (5.2, 20.8)	<0.001

Data source: HINTS 6 (2022). * Models adjusted for age, gender, race/ethnicity, education attainment, employment status, and annual household income. OR: Odds Ratio.

**Table 3:** Crude and adjusted linear regression models estimating the association between interest in a cancer screening test and smoking status.

	Non-Smokers	Former Smokers	Current Smokers
Interested in a cancer screening test			
Mean (95% CI)	2.4 (2.3, 2.5)	2.5 (2.4, 2.6)	2.1 (1.9, 2.3)
Linear regression model *			
Estimate (*p*-value)	Ref	0.05 (*p*-value: 0.4)	−0.2 (*p*-value: 0.05)

Data source: HINTS 6 (2022). * Models adjusted for age, gender, race/ethnicity, education attainment, employment status, and annual household income. Outcome questionnaire: “How interested are you in having a cancer screening test in the next year?”. Response: 1—Not at all; 2—A little; 3—Somewhat; 4—Very.

**Table 4:** Crude and adjusted linear regression models estimating the association between technical problems, quality of care, and privacy concerns with telehealth care by smoking status.

	Non-Smokers Mean (95% CI)	Former Smokers Mean (95% CI)	Current Smokers Mean (95% CI)
Had technical problems	1.6 (1.5, 1.7)	1.5 (1.4, 1.7)	1.6 (1.4, 1.8)
Telehealth as good as in-person	3.0 (2.9, 3.1)	2.9 (2.8, 3.0)	3.4 (3.1, 3.6)
Concerned about the privacy	1.5 (1.4, 1.6)	1.4 (1.3, 1.5)	1.6 (1.2, 2.0)
Model 1 (Crude)		Estimate (*p*-value)	Estimate (*p*-value)
Had technical problems	Ref	−0.03 (*p*-value: 0.6)	0.007 (*p*-value: 0.9)
Telehealth as good as in-person	Ref	−0.08 (*p*-value: 0.3)	0.4 (*p*-value: 0.002)
Concerned about the privacy	Ref	−0.1 (*p*-value: 0.1)	0.07 (*p*-value: 0.7)
Model 2 (Adjusted) *			
Had technical problems	Ref	0.01 (*p*-value: 0.8)	−0.1 (*p*-value: 0.3)
Telehealth as good as in-person	Ref	−0.09 (*p*-value: 0.2)	0.3 (*p*-value: 0.04)
Concerned about the privacy	Ref	−0.02 (*p*-value: 0.7)	−0.1 (*p*-value: 0.2)

Data source: HINTS 6 (2022). * Models adjusted for age, gender, race/ethnicity, education attainment, employment status, and annual household income. Outcome questionnaire: Regarding your telehealth visits, how much do you agree or disagree—I had technical problems with my telehealth visit(s) (for example, difficulty using the technology, trouble seeing or hearing my healthcare provider); The care I received from telehealth was as good as a regular in-person visit; I was concerned about the privacy of my telehealth visit(s). Modified Response: 1—strongly disagree, 2—somewhat disagree, 3—somewhat agree, and 4—strongly agree.

## Data Availability

The HINTS 6 (2022) data are publicly available on the NCI website: https://hints.cancer.gov/ (accessed on 5 June 2024).

## References

[1] CDC Screening for Lung Cancer. https://www.cdc.gov/lung-cancer/screening/index.html.

[2] Aberle D.R., Adams A.M., Berg C.D., Black W.C., Clapp J.D., Fagerstrom R.M., Gareen I.F., Gatsonis C., Marcus P.M., Sicks J.D. (2011). Reduced Lung-Cancer Mortality with Low-Dose Computed Tomographic Screening. N. Engl. J. Med..

[3] Hoffman R.M., Atallah R.P., Struble R.D., Badgett R.G. (2020). Lung Cancer Screening with Low-Dose CT: A Meta-Analysis. J. Gen. Intern. Med..

[4] CDC TobaccoFree Burden of Tobacco Use in the U.S. https://www.cdc.gov/tobacco/campaign/tips/resources/data/cigarette-smoking-in-united-states.html.

[5] US Department of Health and Human Services (2014). The Health Consequences of Smoking—50 Years of Progress: A Report of the Surgeon General.

[6] Cornelius M.E. (2022). Tobacco Product Use Among Adults—United States, 2020. MMWR Morb. Mortal. Wkly. Rep..

[7] CDC Lung Cancer Risk Factors. https://www.cdc.gov/lung-cancer/risk-factors/index.html.

[8] CDC TobaccoFree Diseases/Conditions Featured in the Tips Campaign. https://www.cdc.gov/tobacco/campaign/tips/diseases/index.html.

[9] Hatsukami D.K., Luo X., Jensen J.A., Al’Absi M., Allen S.S., Carmella S.G., Chen M., Cinciripini P.M., Denlinger-Apte R., Drobes D.J. (2018). Effect of Immediate vs Gradual Reduction in Nicotine Content of Cigarettes on Biomarkers of Smoke Exposure: A Randomized Clinical Trial. Jama.

[10] Denlinger-Apte R.L., Kotlyar M., Koopmeiners J.S., Tidey J.W., Luo X., Benowitz N.L., Jensen J.A., Ikuemonisan J.O., Pacek L.R., Smith T.T. (2019). Effects of Very Low Nicotine Content Cigarettes on Smoking Behavior and Biomarkers of Exposure in Menthol and Non-Menthol Smokers. Nicotine Tob. Res..

[11] Hatsukami D.K., Kotlyar M., Hertsgaard L.A., Zhang Y., Carmella S.G., Jensen J.A., Allen S.S., Shields P.G., Murphy S.E., Stepanov I. (2010). Reduced Nicotine Content Cigarettes: Effects on Toxicant Exposure, Dependence and Cessation. Addiction.

[12] Donny E.C., Denlinger R.L., Tidey J.W., Koopmeiners J.S., Benowitz N.L., Vandrey R.G., Al’Absi M., Carmella S.G., Cinciripini P.M., Dermody S.S. (2015). Randomized Trial of Reduced-Nicotine Standards for Cigarettes. N. Engl. J. Med..

[13] Lin W., Krebs N.M., Zhu J., Foulds J., Horn K., Muscat J.E. (2020). Comparison between Gradual Reduced Nicotine Content and Usual Nicotine Content Groups on Subjective Cigarette Ratings in a Randomized Double-Blind Trial. Int. J. Environ. Res. Public Health.

[14] Mercincavage M., Souprountchouk V., Tang K.Z., Dumont R.L., Wileyto E.P., Carmella S.G., Hecht S.S., Strasser A.A. (2016). A Randomized Controlled Trial of Progressively Reduced Nicotine Content Cigarettes on Smoking Behaviors, Biomarkers of Exposure, and Subjective Ratings. Cancer Epidemiol. Biomark. Prev..

[15] Benowitz N.L., Dains K.M., Hall S.M., Stewart S., Wilson M., Dempsey D., Jacob P. (2012). Smoking Behavior and Exposure to Tobacco Toxicants during 6 Months of Smoking Progressively Reduced Nicotine Content Cigarettes. Cancer Epidemiol. Biomark. Prev..

[16] Hammond D., O’Connor R.J. (2014). Reduced Nicotine Cigarettes: Smoking Behavior and Biomarkers of Exposure among Smokers Not Intending to Quit. Cancer Epidemiol. Biomark. Prev..

[17] Foulds J., Veldheer S., Pachas G., Hrabovsky S., Hameed A., Allen S.I., Cather C., Azzouz N., Yingst J., Hammett E. (2022). The Effects of Reduced Nicotine Content Cigarettes on Biomarkers of Nicotine and Toxicant Exposure, Smoking Behavior and Psychiatric Symptoms in Smokers with Mood or Anxiety Disorders: A Double-Blind Randomized Trial. PLoS ONE.

[18] Krebs N.M., Zhu J., Wasserman E., Kuprewicz R., Martinez D.J., Veldheer S., Livelsberger C., Modesto J., Reinhart L., Trushin N. (2021). Switching to Progressively Reduced Nicotine Content Cigarettes in Smokers with Low Socioeconomic Status: A Double-Blind Randomized Clinical Trial. Nicotine Tob. Res..

[19] Lin W., Krebs N.M., Zhu J., Horn K., Foulds J., Evins A.E., Muscat J.E. (2024). Racial Differences in Nicotine Reduction: Pooled Results from Two Double-Blind Randomized Controlled Trials. J. Racial Ethn. Health Disparities.

[20] Almogbel Y. (2020). Smoking Cessation Beliefs Among Saudi University Students in Qassim Region, Saudi Arabia. Risk Manag. Healthc. Policy.

[21] Rutledge C.M., Kott K., Schweickert P.A., Poston R., Fowler C., Haney T.S. (2017). Telehealth and eHealth in Nurse Practitioner Training: Current Perspectives. Adv. Med. Educ. Pract..

[22] Gilman M., Stensland J. (2013). Telehealth and Medicare: Payment Policy, Current Use, and Prospects for Growth. Medicare Medicaid Res. Rev..

[23] Satin A.M., Lieberman I.H. (2021). The Virtual Spine Examination: Telemedicine in the Era of COVID-19 and Beyond. Glob. Spine J..

[24] Garfan S., Alamoodi A.H., Zaidan B.B., Al-Zobbi M., Hamid R.A., Alwan J.K., Ahmaro I.Y.Y., Khalid E.T., Jumaah F.M., Albahri O.S. (2021). Telehealth Utilization during the Covid-19 Pandemic: A Systematic Review. Comput. Biol. Med..

[25] Gajarawala S.N., Pelkowski J.N. (2021). Telehealth Benefits and Barriers. J. Nurse Pract..

[26] Cascella L.M. (2014). Virtual Risk: An Overview of Telehealth from a Risk Management Perspective. https://www.medpro.com/telehealth-onlineprescribing-risks.

[27] Senft Everson N., Jensen R.E., Vanderpool R.C. (2024). Disparities in Telehealth Offer and Use among U.S. Adults: 2022 Health Information National Trends Survey. Telemed. E-Health.

[28] Lucas J.W., Villarroel M.A. (2022). Telemedicine Use among Adults: United States, 2021.

[29] Marbin J., Balk S.J., Gribben V., Groner J., Walley S.C., Boykan R., Jenssen B.P., Mih B., Alfieri N.L., Section on Tobacco Control (2021). Health Disparities in Tobacco Use and Exposure: A Structural Competency Approach. Pediatrics.

[30] Mendez D., Le T.T.T. (2022). Consequences of a Match Made in Hell: The Harm Caused by Menthol Smoking to the African American Population over 1980–2018. Tob. Control.

[31] Bhaskar S., Rastogi A., Menon K.V., Kunheri B., Balakrishnan S., Howick J. (2020). Call for Action to Address Equity and Justice Divide During COVID-19. Front. Psychiatry.

[32] Merianos A.L., Fevrier B., Mahabee-Gittens E.M. (2021). Telemedicine for Tobacco Cessation and Prevention to Combat COVID-19 Morbidity and Mortality in Rural Areas. Front. Public Health.

[33] Miyawaki A., Tabuchi T., Ong M.K., Tsugawa Y. (2021). Age and Social Disparities in the Use of Telemedicine During the COVID-19 Pandemic in Japan: Cross-Sectional Study. J. Med. Internet Res..

[34] Nouri S., Khoong E.C., Lyles C.R., Karliner L. (2020). Addressing Equity in Telemedicine for Chronic Disease Management during the COVID-19 Pandemic. Catal. Innov. Care Deliv..

[35] Jaffe D.H., Lee L., Huynh S., Haskell T.P. (2020). Health Inequalities in the Use of Telehealth in the United States in the Lens of COVID-19. Popul. Health Manag..

[36] Rivera V., Aldridge M.D., Ornstein K., Moody K.A., Chun A. (2021). RESEARCHRacial and Socioeconomic Disparities in Access to Telehealth. J. Am. Geriatr. Soc..

[37] Pierce R.P., Stevermer J.J. (2023). Disparities in the Use of Telehealth at the Onset of the COVID-19 Public Health Emergency. J. Telemed. Telecare.

[38] Darrat I., Tam S., Boulis M., Williams A.M. (2021). Socioeconomic Disparities in Patient Use of Telehealth During the Coronavirus Disease 2019 Surge. JAMA Otolaryngol.–Head Neck Surg..

[39] Westat Health Information National Trends Survey 6 (HINTS 6): Methodology Report National Cancer Institute Bethesda, MD 2024. https://hints.cancer.gov.

[40] Cai J., Bidulescu A. (2023). The Association between E-Cigarette Use or Dual Use of e-Cigarette and Combustible Cigarette and Prediabetes, Diabetes, or Insulin Resistance: Findings from the National Health and Nutrition Examination Survey (NHANES). Drug Alcohol Depend..

[41] Jamal A. (2018). Current Cigarette Smoking among Adults—United States, 2016. MMWR Morb. Mortal. Wkly. Rep..

[42] Lin W. (2024). Association Between Time to First Cigarette Use and Urine Biomarkers of Tobacco Exposure in Adult Smokers. Lung.

[43] Lin W., Zhu J., Hayes J.E., Richie J.P., Muscat J.E. (2022). Comparison of Carcinogen Biomarkers in Smokers of Menthol and Nonmenthol Cigarettes: The 2015–2016 National Health and Nutrition Examination Survey Special Sample. Cancer Epidemiol. Biomark. Prev..

[44] Lin W., Muscat J.E. (2021). Knowledge and Beliefs Regarding Harm from Specific Tobacco Products: Findings from the HINT Survey. Am. J. Health Promot..

[45] American Lung Association New Report: Critically Low Lung Cancer Screening Rates Reveal Opportunity to Save More Lives. https://www.lung.org/media/press-releases/state-of-lung-cancer-2022.

[46] American Lung Association (2022). State of Lung Cancer 2022. https://www.lung.org.

[47] CDC Lung Cancer Statistics. https://www.cdc.gov/lung-cancer/statistics/index.html.

[48] Cataldo J.K. (2016). High-Risk Older Smokers’ Perceptions, Attitudes, and Beliefs about Lung Cancer Screening. Cancer Med..

[49] Siegel R.L., Miller K.D., Fuchs H.E., Jemal A. (2021). Cancer Statistics, 2021. CA Cancer J. Clin..

[50] Centers for Disease Control and Prevention (US), National Center for Chronic Disease Prevention and Health Promotion (US), Office on Smoking and Health (US) (2010). How Tobacco Smoke Causes Disease: The Biology and Behavioral Basis for Smoking-Attributable Disease: A Report of the Surgeon General.

[51] 2001 Surgeon General’s Report|Women and Smoking|Smoking & Tobacco Use|CDC. https://archive.cdc.gov/www_cdc_gov/tobacco/sgr/2001/index.htm.

[52] Cornelius M.E. (2023). Tobacco Product Use Among Adults—United States, 2021. MMWR Morb. Mortal Wkly. Rep..

[53] Hawk E.T., Colbert Maresso K. (2019). E-Cigarettes: Unstandardized, Under-Regulated, Understudied, and Unknown Health and Cancer Risks. Cancer Res..

[54] Løchen M.-L., Gram I.T., Mannsverk J., Mathiesen E.B., Njølstad I., Schirmer H., Wilsgaard T., Jacobsen B.K. (2017). Association of Occasional Smoking with Total Mortality in the Population-Based Tromsø Study, 2001–2015. BMJ Open.

